# Distribution and Radiosensitizing Effect of Cholesterol-Coupled Dbait Molecule in Rat Model of Glioblastoma

**DOI:** 10.1371/journal.pone.0040567

**Published:** 2012-07-17

**Authors:** Nicolas Coquery, Nicolas Pannetier, Régine Farion, Aurélie Herbette, Leire Azurmendi, Didier Clarencon, Stéphane Bauge, Véronique Josserand, Claire Rome, Jean-Luc Coll, Jian-Sheng Sun, Emmanuel L. Barbier, Marie Dutreix, Chantal C. Remy

**Affiliations:** 1 Inserm, U836, Grenoble, France; 2 Université Joseph Fourier, Grenoble Institut des Neurosciences, UMR-S836, Grenoble, France; 3 DNA Therapeutics, Evry, France; 4 Institut de Recherche Biomédicale des Armées, Antenne de La Tronche, Centre de Recherches du Service de Santé des Armées, France; 5 Inserm, U823, Grenoble, France; 6 Université Joseph Fourier, Institut Albert Bonniot, UMR-S823, Grenoble, France; 7 Muséum National d’Histoire Naturelle, USM503, Paris, France; 8 Institut Curie Hospital, CNRS UMR3347, INSERM U2021, Orsay, France; University of Manchester, United Kingdom

## Abstract

**Background:**

Glioma is the most aggressive tumor of the brain and the most efficient treatments are based on radiotherapy. However, tumors are often resistant to radiotherapy due to an enhanced DNA repair activity. Short and stabilized DNA molecules (Dbait) have recently been proposed as an efficient strategy to inhibit DNA repair in tumor.

**Methodology/Principal Findings:**

The distribution of three formulations of Dbait, (i) Dbait alone, (ii) Dbait associated with polyethylenimine, and (iii) Dbait linked with cholesterol (coDbait), was evaluated one day after intratumoral delivery in an RG2 rat glioma model. Dbait molecule distribution was assessed in the whole organ with 2D-FRI and in brain sections. CoDbait was chosen for further studies given its good retention in the brain, cellular localization, and efficacy in inducing the activation of DNA repair effectors. The radiosensitizing effect of coDbait was studied in four groups of rats bearing RG2-glioma: no treatment, radiotherapy only, coDbait alone, and CoDbait with radiotherapy. Treatment started 7 days after tumor inoculation and consisted of two series of treatment in two weeks: coDbait injection followed by a selective 6-Gy irradiation of the head. We evaluated the radiosensitizing effect using animal survival, tumor volume, cell proliferation, and vasculature characteristics with multiparametric MRI. CoDbait with radiotherapy improved the survival of rats bearing RG2-glioma by reducing tumor growth and cell proliferation without altering tumor vasculature.

**Conclusion/Significance:**

coDbait is therefore a promising molecular therapy to sensitize glioma to radiotherapy.

## Introduction

Among all the tools available to clinicians, radiotherapy-based treatments are currently the leading strategy used to improve the clinical outcome in glioma patients [1]. However, several limitations are often encountered after irradiation, such as necrosis in normal brain tissue and, importantly, the capability of tumor cells to develop molecular mechanisms leading to radioresistance [2].

One approach to improve patients’ outcome is to combine the beneficial effect of radiotherapy (RT) with the effects of already clinically approved therapies, such as antiangiogenic, vascular disruptive agents, and cytotoxic agents [3]–[5]. Another emerging strategy is to directly target the mechanisms that are involved in radioresistance [2]. Given that irradiation directly damages DNA, mainly in the form of single- or double-strand breaks (DSB) [6], several molecules are currently in preclinical or clinical trials and aim at inhibiting proteins that are involved in one of the DNA repair pathways [7], [8]. However, to date, none of these molecular therapies has proved to be successful. Here we have tested a new strategy for inhibiting the DSB repair pathway called Dbait in association with RT for treating glioblastoma in an orthotopic animal model.

Dbait molecules are short double-strand DNA that mimic DSB and are recognized as damaged by repair and signaling proteins. As a consequence, they trigger a “false” signaling of DNA damage and inhibit repair of the damage induced by irradiation in treated cells [9]. This approach has already given promising results in radiosensitizing several types of tumors *in vitro* and *in vivo* in subcutaneous xenograft models [10], [11]. The preclinical studies on skin melanoma indicate that Dbait is well tolerated and does not increase radiotoxicity on healthy skin. These results led us to start a phase I clinical trial to assess the tolerance and efficacy of Dbait combined with RT on patients with melanoma in transit (#NCT01469455, registered on http://clinicaltrials.gov).

The Dbait strategy has not been evaluated in an orthotopic model of glioma. Since irradiation-induced central nervous system neurotoxicity is a major problem in developing new therapeutic approaches for treating glioblastoma, it is important to assess its safety and efficacy on animals using Dbait to improve RT in brain tissue. Dbait molecules do not spontaneously enter cells of subcutaneous tumors [11]. Several publications have described efficient cellular uptake in mouse brain of naked DNA [12] or DNA combined with PAMAM dendrimer conjugates [13]–[15], lipids [16], [17], or polyethylenimine [16], [18]. Dbait molecules were previously administered in subcutaneous tumors after complexion with polyethylenimine (PEI), which increases intracellular delivery [10]. However, the range of Dbait concentrations achievable in association with PEI remains limited for *in vivo* purposes. This limitation can be overcome using cholesterol as a transfection agent. This approach has already shown efficiency for *in vivo* delivery of siRNA in the central nervous system [19], and recently of Dbait molecules in zebrafish early embryos and in mouse xenografted tumors [11].

Here, we first studied the distribution of various Dbait formulations after injection into healthy striatum or into tumor tissue in the RG2 orthotopic rat model of glioma. We then assessed the safety of cholesterol-coupled Dbait (coDbait) injection with RT in normal rat brain. The radiosensitizing effect of coDbait was finally addressed by evaluating the survival of rats bearing RG2-glioma and by analyzing tumor growth and microvasculature with multiparametric MRI [20], [21].

## Methods

### Ethic Statement

All animal experiments were conducted under permits no. 380820 and no. A3851610008 from the French Ministry of Agriculture. The protocols were approved by the local committee for animal care and use of the Grenoble Institute of Neuroscience (Comité d’éthique pour l’expérimentation animale, GIN, amended by the Comité National de Réflexion Ethique sur l’Expériemtation Animale). All surgery was performed under isoflurane anesthesia with additional local analgesia, and all efforts were made to minimize suffering.

### Animal Model

The RG2 cells (ATCC, CRL-223) were implanted in the brain of male Fisher 344 rats (150–200 g, Charles River, France) with a stereotactic frame as previously described [22]. Tumor cells were inoculated under anesthesia with the following parameters: 5% isoflurane for induction and 2.5% for maintenance in 100% air. Bupivacaine (8 mg/kg) was injected subcutaneously before incision to prevent postoperative pain. RG2 cells (5 10^3^) in 5 µL serum-free alpha-MEM medium were inoculated at 5 µL/min in the right caudate nucleus through a 1-mm burr hole with the following coordinate from Bregma: AP = 0, ML = 3.5, DV = 5.5 mm. After injection, the burr hole was filled with bone wax, the skin incision was sewed and the animals were observed until awakening prior to being returned to the animal facility.

### Treatments

#### Dbait molecules

Dbait32Hc (molecular mass, 20.153 kDa) molecules were obtained by automated solid-phase oligonucleotide synthesis from Eurogentec (Seraing, Belgium). Three formulations of Dbait were used: Dbait32Hc alone (Dbait), Dbait32Hc in a mixture with in vivo-jet polyethylenimine reagent (PEI; Polyplus Transfection) (Dbait/PEI) as previously described [10], and Dbait32Hc coupled with a molecule of cholesterol (coDbait). For the distribution analysis, Dbait molecules were additionally coupled with the near infra-red (NIR) cyanine-5.5 dye. All Dbait formulations were diluted in a solution of 5% glucose.

#### Dbait formulation delivery and quantities

Intracranial and intratumoral injections were made by convection enhanced delivery (CED). This procedure consists of an injection at a limited rate (0.5 µL/min) of 10 µL for a duration of 20 min. The CED procedure ensures a homogenous deposit of a high amount of molecules into the targeted tissue. Animals were placed in a stereotactic frame and the operation was performed as described above. For distribution studies, the quantities of Dbait molecules were the following: Dbait alone and coDbait: 100 µg, Dbait/PEI: 10 µg. The quantity of Dbait/PEI used was the maximum achievable quantity of the Dbait molecule and dictated by Dbait solubility in the PEI solution. Two injections of coDbait, 100 µg each, were made to assess the radiotherapeutic protocol in non tumoral rat brain. At the same time, the brain tolerance study was conducted using two quantities of coDbait: 200 and 500 µg. The radiosensitizing effect was evaluated on tumoral brain with two injections of coDbait, 400 µg each.

#### Chloroquine injection

Chloroquine was used for its ability to promote entry of DNA molecules into the cell cytoplasm via increased endocytosis [23]. Chloroquine (Sigma) was prepared at 10 mg/ml in saline solution and was used either freshly or after storage at 4°C for up to 2 days. Chloroquine (10 mg/kg) was administered with 2 intraperitoneal (ip) injections 1 day and at least 3 hours prior to the administration of Dbait molecules.

#### Irradiation

Isoflurane-anesthetized rats received gamma irradiation (^60^Co, source ICO 4000) [24] at a dose rate of 0.50 Gy/min. The source of irradiation was collimated in order to specifically target the animals’ head. Control rats were anesthetized but were not exposed to the radiation source.

### Dbait Quantification

Brain samples were weighed and homogenized with lysis buffer (guanidium hydrochloride, sodium acetate, lauroylsarcosine; 5 mL of lysis buffer per gram of tissue) using a tissue homogenizer. The concentration of coDbait in tissue samples was quantified by a specific hybridization immunoassay with the following protocol. The samples were then incubated at 95°C for 12 min and cooled to room temperature for 20 min to form a duplex with 300 µL of the capture probe (5′−/5BioTEG/GCT GTG CCC ACA ACC -3′) at 20 nM in 5× SSCT (0.75 M NaCl, 0.075 M sodium citrate, 0.01% Tween-20). We then added 100 µL of this mixture in duplicate to the wells of a 96-well streptavidin-coated plate (StreptaWell, Roche Applied Science). The plate was covered and incubated for 45 min at room temperature on an orbital shaker set at 200 rpm and washed three times with 2× SSCT (300 mM NaCl, 30 mM sodium citrate, 0.01% Tween-20). A detection probe (5′- CAG CAA ACA AGC CTA GA/3DigN/−3′) at 20 nM in 5× SSCT was added to all wells (100 µL). The plate was incubated for 45 min at room temperature on an orbital shaker set at 200 rpm, washed three times with 2× SSCT, and incubated 45 min at room temperature with an anti-digoxigenin peroxidase conjugated antibody (Roche Applied Science) diluted 1∶10,000 in PBS; 0.005% Tween-20 was added to all wells (100 µL). Detection was achieved by the addition of a TMB substrate (100 µL) for approximately 45 min at room temperature and stopped by the addition of H2SO4 0.5 M (100 µL). The absorbance was measured using a Spectramax 340PC or Plus 384 microplate reader (Molecular Devices Corporation) set at 450 nm with a 570-nm background correction. The amount of coDbait in samples was calculated from the calibration standards over a working range of 3.00–80.00 ng/mL using a four-parameter logistic curve fit.

### Multiparametric MR Imaging and Analysis

MRI was performed at 4.7 T (Avance III console; Bruker, Grenoble Preclinical MRI facility). A T2-weighted (T_2_W) sequence (TR/TE = 4000/33 ms, voxel size = 117×117×1000 µm) was used to determine tumor volume and delineate the two regions of interest (ROIs: tumor and contralateral striatum) used for the MRI parameters. The apparent diffusion coefficient (ADC) was mapped (TR/TE = 3000/28.6 ms, b≈0 and b = 900 s/mm^2^, voxel size = 234×234×1000 µm). Vascular integrity was assessed using a dynamic contrast- enhanced MRI approach as previously described [21]. Briefly, multiple T1-weighted images (n = 60, 15.6 s per image) were acquired (TR/TE: 800/4.2 ms, voxel size = 234×234×1000 µm). After acquisition of ten baseline images, a bolus of Gd-DOTA (200 µmol/kg, flushed with 250 µL of saline) was administered through the tail vein. The area under the curve of Gd-DOTA (AUC_Gd-DOTA_) was calculated using the 40 consecutive images subtracted from baseline. The blood volume fraction (BVf) and vessel size index, related to the vessel diameter (VSI) [25] were obtained from a second bolus injection using a first-passage approach with a spiral gradient echo, spin echo sequence (2 shots, TR/GETE/SETE:250/16.5/50.1 ms, voxel size = 375×375×1000 µm) [26]. To normalize the BVf values obtained in each animal, the BVf in the contralateral striatum was set at 3% [22]. All parametric maps were computed using software developed in our laboratory with Matlab (MathWorks, Natick, MA, USA).

### Two Dimensional-fluorescence Reflectance Imaging

Organ distribution of Dbait-Cy5.5 formulations was performed with 2 dimensional-fluorescence reflectance imaging (2D-FRI) system (Fluobeam) as previously described [27]. Briefly, the system is composed of 2 optical fibers 690-nm emitting lasers (100 mW each) providing NIR excitation and a charge-coupled device (CCD) camera to detect fluorescence emitted by Cy5.5. This system provides continuous NIR excitation because of the presence of a holographic lens that produces a homogeneous lightened field (6–8 cm in diameter) with an illumination power of 5 mW/cm^2^. The fluorescence signal is collected through a high-pass filter (>720 nm) by a digital 12-bit CCD camera that records time-lapse sequences of images similar to a video rate (image size, 1392×1024; pixel size, 6.45×6.45 µm^2^). The fluorescent images were acquired in 5 ms and were superimposed automatically on a visible light image obtained using an infrared-filtered white-light illuminator (7×10^3^ lux). The brains were imaged separately, whereas all the organs for each animal were imaged together. For the quantification of fluorescence, expressed in mean number of photons per pixel, the signal was corrected to the initial quantity of injected fluorescent molecules and normalized to 1 ms. Given that cholesterol and PEI molecules are able to locally quench the fluorescence emitted by the Cy5.5 dye when associated with Dbait, the measurement of fluorescence was corrected with a previously determined coefficient (Dbait = 1, coDbait = 1.73, Dbait/PEI = 3.13) [11]. Quantification is thus related to the same amount of injected fluorescence between formulations.

### Brain Sectioning and Histology

Coronal cryosections (10 µm thick) were cut along the entire striatum or tumor on a microtome (Leica). Hematoxylin-erythrosine staining was used as previously described after fixing in methanol/acetone (50%, v/v) and washing in tap water for 5 min [28]. Histological observation using NIR imaging of Cy5.5-labeled formulations of Dbait used the MacroFluo macroscope (Leica) and the LSM710 confocal microscope (Zeiss). For the immunohistology of p-RPA32, primary antibody was rabbit polyclonal anti-RPA32 ser4/ser8 NB100-543 diluted 1∶100 (NB 100*–*543, Novus Biologicals) and secondary antibody was anti-rabbit Alexa 488 diluted 1∶200 (Invitrogen).

For the immunohistology of Ki67, MBP, and ED1, the brains were fixed in neutral buffered formalin, embedded in paraffin. Coronal cryosections (7 µm thick) were cut along the entire tumor. Primary antibodies were rabbit polyclonal anti-Ki67 diluted 1∶500 (Ab15580, Abcam), mouse anti-ED1 diluted 1∶1,000 (AbC117-6714, AbCys), and rat anti-MBP diluted 1∶100 (VMA386, AbCys). Secondary biotin-conjugated antibodies were goat anti-rabbit diluted 1∶200 (BA-1000, Vector Laboratories), donkey anti-mouse diluted 1∶1000 (SC-2098, Santa Cruz), and rabbit anti-rat 1∶500 (BA-4000, Vector Laboratories). Extradivin-conjugated peroxidase was added 1∶1000 (E-2886, Sigma) and detected with the DAB Peroxidase Substrate Kit (SK-4100, Vector Laboratories).

### Statistical Analysis

The number of rats for each experiment is given in the figure legends. The log-rank test was used to detect statistical significance in survival. Analysis of variance (ANOVA) was performed to detect statistical significance in the measurement of coDbait-Cy5.5 in various organs. The Student *t*-test was used to detect an effect of coDbait on the number of Ki67-positive tumor cells. Repeated Measurement of ANOVA was performed to detect significant effects of coDbait, RT, or combined treatments on tumor growth and vascular properties determined with MRI. A p-value <0.05 was considered as significant.

## Results

### Distribution of Dbait, Dbait/PEI, and coDbait 1 day after Intrastriatal Injection in Normal Rats

**Figure 1:**
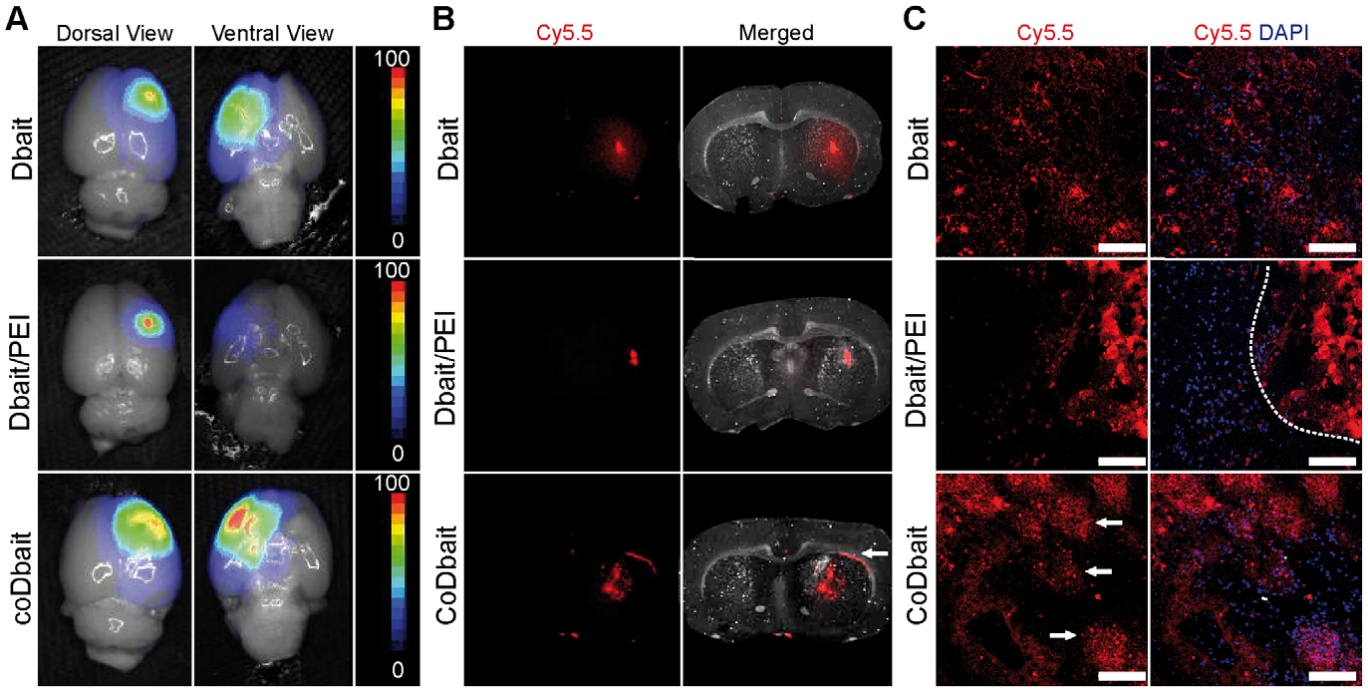
Distribution in normal brain of various formulations of Dbait, 1 day after intrastriatal injection. Rats received an intracranial injection by CED of one Dbait formulation coupled with the Cy5.5 near-infrared dye (Dbait-Cy5.5∶100 µg, n = 2; Dbait-Cy5.5/PEI: 10 µg, n = 2; coDbait-Cy5.5∶100 µg, n = 2). (A) Distribution in whole brain (dorsal and ventral views) was determined with 2D-FRI. Intensity scale was normalized within each group. (B) Distribution in brain sections determined with macroscopy. The white arrows show a local accumulation of coDbait in corpus callosum. (C) Distribution at the cellular level determined with confocal microscopy. The dotted line in the Dbait/PEI panel delineates the local deposit near the needle track and the white arrows show a local accumulation of coDbait in striosomes determined with light microscopy. Scale bar = 100 µm.

We first investigated the distribution in normal brain tissue of various Dbait formulations 1 day after intrastriatal injection. This duration has been proven to be necessary for an effective sensitizing effect of Dbait molecules to RT [10], [11]. As in the Berthault et al. study [11], the Dbait molecules were linked to Cy5.5; the quantity of Dbait alone and coDbait was chosen to avoid fluorescence signal saturation. Due to the solubility limit of the Dbait molecule when associated with PEI, the maximum achievable quantity was 10 µg of Dbait molecules. Observation of 2D-FRI on dorsal and ventral views of the brain showed a large distribution of coDbait-Cy5.5 and Dbait-Cy5.5 within the ipsilateral hemisphere, whereas the signal from Dbait-Cy5.5/PEI was localized in the vicinity of the needle tract (Fig. 1A). These observations were validated with macroscopic analysis (Fig. 1B). In addition, we detected a high affinity of coDbait-Cy5.5 with white matter structures such as the corpus callosum (Fig. 1B, white arrow) and striosomes. This affinity of coDbait-Cy5.5 for striosomes was confirmed with confocal microscopy (Fig. 1C, white arrows). Dbait-Cy5.5 was found either concentrated along the needle track or diffused in brain tissue with no specific localization. A local deposit of Dbait-Cy5.5/PEI was confirmed with little diffusion in surrounding tissue (Fig. 1C, dotted line).

### Distribution and Effect of Dbait, Dbait/PEI, and coDbait 1 day after Intratumoral Injection in RG2 Glioma-bearing Rats

**Figure 2:**
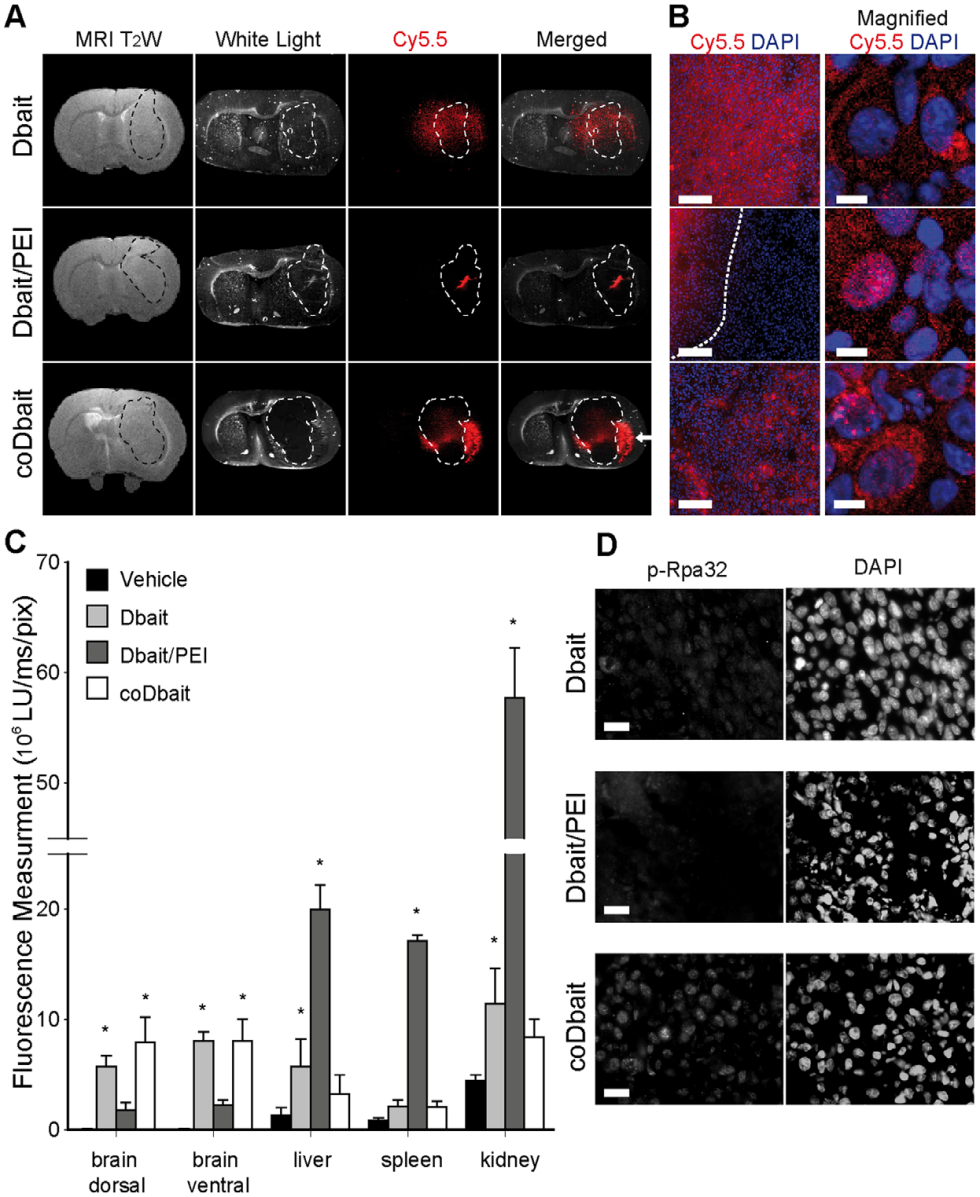
Distribution, quantification, and effect of various formulations of Dbait 1 day after intratumoral injection in RG2 glioma. (A–D) Rats bearing a 14-day-old tumor received an intratumoral injection by CED of one Dbait formulation coupled with the near-infrared dye Cy5.5 (Dbait-Cy5.5∶100 µg, n = 4; Dbait-Cy5.5/PEI: 10 µg, n = 4; coDbait-Cy5.5∶100 µg, n = 4). Intratumoral injection was guided with anatomic MRI performed 1 day prior to injection. Images from one animal representative of each group are presented. (A) Anatomic localization of RG2 tumor with T_2_W MRI and corresponding distribution of Dbait formulations in brain sections determined with macroscopy. The white arrows show a local accumulation of coDbait in corpus callosum. (B) Distribution of Cy5.5 at the cellular level determined with confocal microscopy; left panel scale bar = 100 µm and right panel scale bar = 6.7 µm. The dotted line in the Dbait/PEI panel delineates the local deposit near the needle track. (C) Fluorescence measurement of Cy5.5 in several organs determined with 2D-FRI and compensated for the quantity of fluorescent molecules injected. Value expressed as light unit (LU) per ms and pixel. ANOVA, Dbait formulations versus vehicle, *: p<0.05. Mean ± SD. (D) Immunodetection of p-Rpa32, scale bar = 20 µm.

We next investigated the distribution of the various formulations of Dbait after intratumoral injection in the RG2-glioma model (Fig. 2). Dbait-Cy5.5 and coDbait-Cy5.5 signals were diffused inside and outside the tumor bulk, whereas Dbait-Cy5.5/PEI was localized near the injection track with little diffusion in surrounding tissue (Fig. 2A and 2B). The signal from coDbait-Cy5.5 was also found to be intense at the tumor border and to some extent along white matter structures in proximity of the tumor (Fig. 2A, white arrow), which corroborates the previous observations after intrastriatal injection (Fig. 1B).

High magnification of confocal microscopy showed coDbait-Cy5.5 localized in the nucleus and cytoplasm, Dbait-Cy5.5 mainly localized in the extracellular space, and a nuclear distribution for Dbait-Cy5.5/PEI (Fig. 2B, right panel). Similar to Chen et al. [19], we observed a selective fixation of cholesterol-coupled DNA molecules on the white matter track and presumably on myelin sheets. All these observations are in accordance with the recent study of Berthault et al. [11], which explored the distribution and toxicity of various Dbait formulations on zebrafish embryos.

The distribution in the brain determined with 2D-FRI was similar to the distribution after intrastriatal injection (data not shown), which validates the efficacy of CED. We also used the 2D-FRI to quantify the florescence in various organs after isolation (Fig. 2C). The level of Cy5.5 in the brain was significantly higher after Dbait-Cy5.5 and coDbait-Cy5.5 injection in comparison with control vehicle injection, whereas no statistical difference was detected after Dbait-Cy5.5/PEI injection. A strong signal was detected in liver, spleen, and kidney after Dbait-Cy5.5/PEI injection but was much lower after Dbait-Cy5.5 injection and even lower after coDbait-Cy5.5 injection. The presence of Dbait molecules in these organs might suggest that that they could also be found in proliferating tissues such as gut and bone marrow. We did not measure the amount of coDbait molecule in these organs. However, toxicity studies on rats and monkeys show that coDbait is well tolerated with a no-observed-adverse-effect level (NOAEL) considered to be the highest tested dose of 32 mg/animal/day (dose range, 161−216 mg/kg/day for rats and 10.7−16.2 mg/kg/day for monkeys) (Schlegel et al., 2012, in press [29]). Moreover, Dbait molecules mainly exert their antiproliferative action upon irradiation [10]. As we chose a selective irradiation protocol that targeted the animal’s head, we can assume a minor effect of Dbait molecules in other organs than the brain.

To confirm that Dbait molecules have reached their main target, the DSB repair enzyme DNA-PK, and to promote their activation, we monitored the phosphorylated form of Rpa32 with immunodetection [9]. Tumors treated by coDbait contained a high number of p-Rpa32-positive cells (Fig. 2D), whereas very few were detected after Dbait and Dbait/PEI injection. Comparative studies with naked Dbait or Dbait formulated with PEI indicate that coDbait displays good retention in brain, preferential distribution in tumor cells, and a potent effect on DNA repair activation.

Before choosing coDbait as the therapeutic form for evaluating an effect of Dbait molecules on sensitizing tumor to RT in animal models, we quantified coDbait concentration in various brain areas 1 day after intratumoral injection. Rats bearing a 14-day-old tumor received an intratumoral injection by CED of coDbait (400 µg, n = 3). Brain samples were isolated and homogenized and Dbait content was measured. For this study, we used a new technology based on hybridization combined with ELISA detection (see Material and Methods) that specifically quantifies coDbait molecules with the complete DNA sequence. As previously observed by fluorescence analysis, the concentration of coDbait was very low in the contralateral (0.8±0.8 µg/g of tissue) and ipsilateral (30.5±21.8 µg/g of tissue) brain hemispheres in comparison with the level of coDbait found in tumor bulk (305.5±277.9 µg/g of tissue). Note that coDbait level achieved its maximum in the peritumoral area (673.6±213.0 µg/g of tissue).

### Tolerance to coDbait in Normal Brain

**Figure 3:**
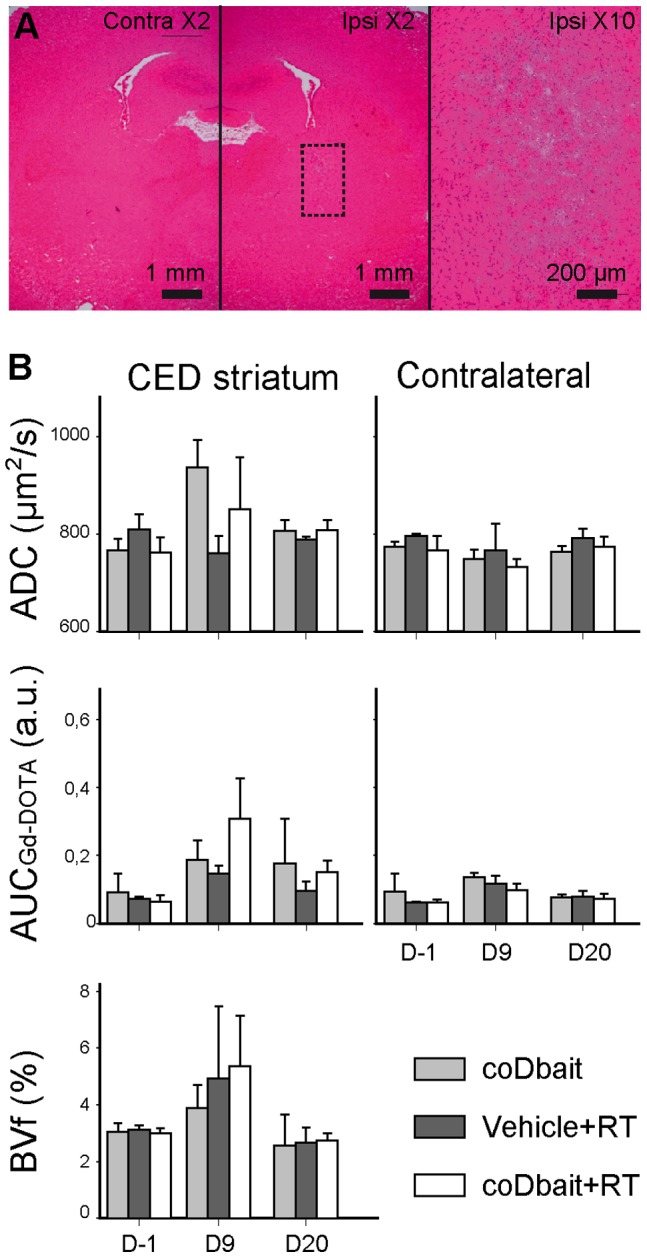
Long-term effect of coDbait in normal brain. (A) Hematoxylin-eosin stained sections of normal brain, 14 days after injection in striatum of coDbait (500 µg). Rats received an intracranial injection by CED of coDbait (200 and 500 µg, n = 2 each). Rats were euthanized 7 or 14 days after injection and brain sections were observed with histology after hematoxylin-erythrosine histology. Contra: contralateral hemisphere, ipsi: ipsilateral injected hemisphere. Dotted rectangle represents the area of the ×10 magnification. (B) Effect of coDbait and RT in normal brain on the injected and contralateral striatum properties determined with multiparametric MRI. Treatment was organized as follows: D0: intratumoral injection by CED of coDbait (100 µg) or vehicle (glucose 5%), D1∶6-Gy irradiation, D6: same as D0, D7: same as D1, see Fig. 5A for a more concise view of the experimental set-up. Three combinations of treatments were administered: coDbait, vehicle+RT, and coDbait+RT (n = 3 each). The animals’ weight was monitored. Multiparametric MRI was performed before (D-1) treatment, 2 days (D9) and 13 days (D20) after treatment completion. ADC: apparent diffusion coefficient, AUC_Gd-DOTA_: area under curve of Gd-DOTA extravasation, BVf: blood volume fraction. Mean BVf in the contralateral striatum was set at 3% for normalization purposes. Mean ± SD.

We controlled animal and tissue tolerance to coDbait 7 and 14 days after intrastriatal injection. For this purpose, we used 200 and 500 µg of coDbait in order to determine tolerance with a large quantity of Dbait molecules. The animals showed no sign of behavioral deficit, based on clinical observation, and had a normal weight curve. Histological observations 7 and 14 days after injection of 200 and 500 µg of coDbait were similar and confirmed that the structure of brain parenchyma is preserved (Fig. 3A). We observed a minor loosening of tissue that might be due to coDbait binding to white matter tracts, as seen in Fig. 1B and 2A, and/or to the mode of injection given that a similar aspect was seen when no solution or only the vehicle was injected (data not shown).

### The Experimental Setup Consisting of 2 coDbait Injections Each Followed by a 6-Gy Irradiation is well Tolerated in Normal Rats

Our main goal is to amplify the positive effect of RT and simultaneously reduce the deleterious effects on surrounding tissue. Therefore, we combined injection of coDait 1 day before each RT treatment (two treatment cycles over 1 week) to minimize the invasiveness of the procedure with repeated anesthesia. For this preliminary experiment, we chose a well-tolerated quantity of coDbait: 100 µg for each injection. Two irradiation protocols were tested on normal rats, consisting of (i) a 2×6-Gy RT: one 6-Gy session after each coDbait injection (n = 12 rats) or (ii) a 4×5-Gy RT: two consecutive 5-Gy sessions over 1 day after each coDbait injection (n = 12 rats). For each protocol, three treatment combinations were tested: coDbait, vehicle+RT, and coDbait+RT. Independently of the coDbait injection, most animals that received the 4×5-Gy RT lost more than 20% of their normal weight, suggesting low tolerance of the irradiation protocol, which forced us to abort the experiment. This was probably due to the repeated anesthesia and irradiation. For the groups that were exposed to the 2×6-Gy RT, the animals’ weight decreased slightly (less than 10%) during treatment and increased after its completion (data not shown).

For the 2×6-Gy RT protocol, we also looked at the effect of treatment on brain tissue properties assessed by multiparametric MRI before (D-1) treatment, 2 days (D9) and 13 days (D20) after treatment completion (Fig. 3B). Compared to D-1, no effect was detected in the contralateral striatum. Some trends were observed at D9 on ADC, BVf, and AUC_Gd-DOTA_ in the injected hemisphere; however, none of them was statistically significant and they returned to the baseline level on D20. All these data indicate that the 2×6-Gy RT protocol was well tolerated and can be applied to glioma-bearing rats.

### coDbait with RT Increases the Animals’ Lifespan by Reducing Tumor Progression without Impacting Tumor Vascularization

**Figure 4:**
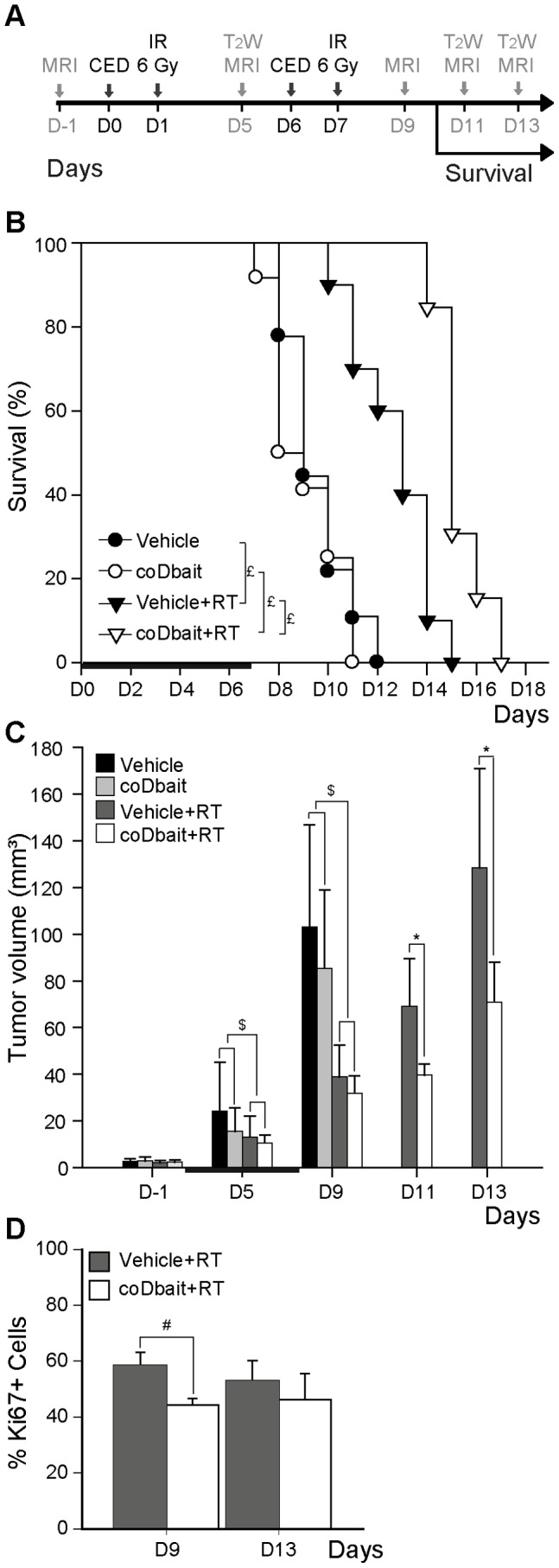
Effect of treatments on animal survival, tumor growth, and cell proliferation. (A) Experimental design**,** animals (n = 68) were implanted with RG2 glioma 14 days prior to treatment. Treatment was organized as follows: D0: intratumoral injection of coDbait (400 µg) or vehicle (glucose 5%), D1∶6-Gy irradiation, D6: same as D0, D7: same as D1. Four combinations of treatments were performed: vehicle, coDbait, vehicle+RT, and coDbait+RT. The animals were euthanized at the onset of neurologic signs [42]. The day of euthanasia was recorded to monitor the effect of treatments on survival. MRI was performed as follows: D-1: multiparametric MRI and group randomization based on tumor volume, D5: anatomic MRI, D9: multiparametric MRI, D11 and D13: anatomic MRI. This experiment was carried out in two studies that reproduced a similar tumor growth pattern for each group so that the data could be pooled. The first study was done to assess animal survival, whereas the second one was done to assess tumor progression after D9 and to perform the histological analysis on brain tissues isolated at D11 and D13. (B) Kaplan-Meier survival plot. The horizontal bar depicts treatment duration. Log rank test, £: p<0.05 (vehicle n = 9; coDbait n = 12; vehicle+RT n = 10; coDbait+RT n = 13). (C) Tumor volume measured with T_2_W MRI before (D-1; vehicle n = 12; coDbait n = 6; vehicle+RT n = 16; coDbait+RT n = 17), during (D5; vehicle n = 12; coDbait n = 6; vehicle+RT n = 16; coDbait+RT n = 17), and after treatment (D9: vehicle n = 12; coDbait n = 6; vehicle+RT n = 16; coDbait+RT n = 17; D11 and D13: vehicle n = 5; vehicle+RT n = 7; coDbait+RT n = 14). The horizontal black bar depicts treatment duration. RM-ANOVA, $: effect of RT between D-1 and D5 or D-1 and D9, p<0.05, *: effect of coDbait with RT between D-1 and D11 or D-1 and D13, p<0.05. (D) Percentage of Ki67-positive cells in tumor bulk at D9 (vehicle+RT n = 6; coDbait+RT n = 4) and D13 (vehicle+RT n = 8; coDbait+RT n = 4). Student *t*-test, #: effect of coDbait with RT. Mean ± SD.

The experimental protocol used to assess the radiosensitizing effect of coDbait is presented in Figure 4A. Two coDbait injections, 400 µg each, were performed at 1-week intervals. One day after each coDbait injection, a 6-Gy irradiation was applied to the whole brain. The quantity of coDbait molecules was chosen slightly lower than the maximal dose tested in the tolerance study (500 µg) to minimize the potential effect of two repeated injections.

The good tolerance of animals to coDbait treatment was confirmed with animal survival that did not differ between untreated animals and animals that received coDbait (Fig. 4B). In contrast, Kaplan-Meier survival analysis revealed a statistically significant prolongation of animal survival in the vehicle+RT group (median, 22 days) compared with the vehicle and coDbait groups (median, 18 versus 17 days, respectively). This prolongation was significantly increased in the coDbait+RT group (median, 24 days) in comparison with the vehicle+RT group.

To confirm that survival extension was the consequence of better tumor growth control by the combined treatment, we monitored tumor progression, as shown in Figure 4C, with T_2_W anatomic MRI before (D−1), during treatment (D5), and at three time points after the end of treatment (D9, D11, and D13). At early times, RM-ANOVA between D-1 and D9 revealed a significant reduction in tumor growth induced by RT, but no additional effect of coDbait was detected. However, after D9, RM-ANOVA revealed that the tumor progression was statistically delayed with coDbait+RT treatment in comparison with vehicle+RT treatment. Consistent with this finding, histological analysis of the tumors confirmed that the amount of proliferating Ki67-positive cells was significantly reduced on D9 after coDbait+RT treatment as compared to single RT (Fig. 4D). Interestingly, this difference decreased at D13 as tumors started to grow again (D13, Fig. 4C).

**Figure 5:**
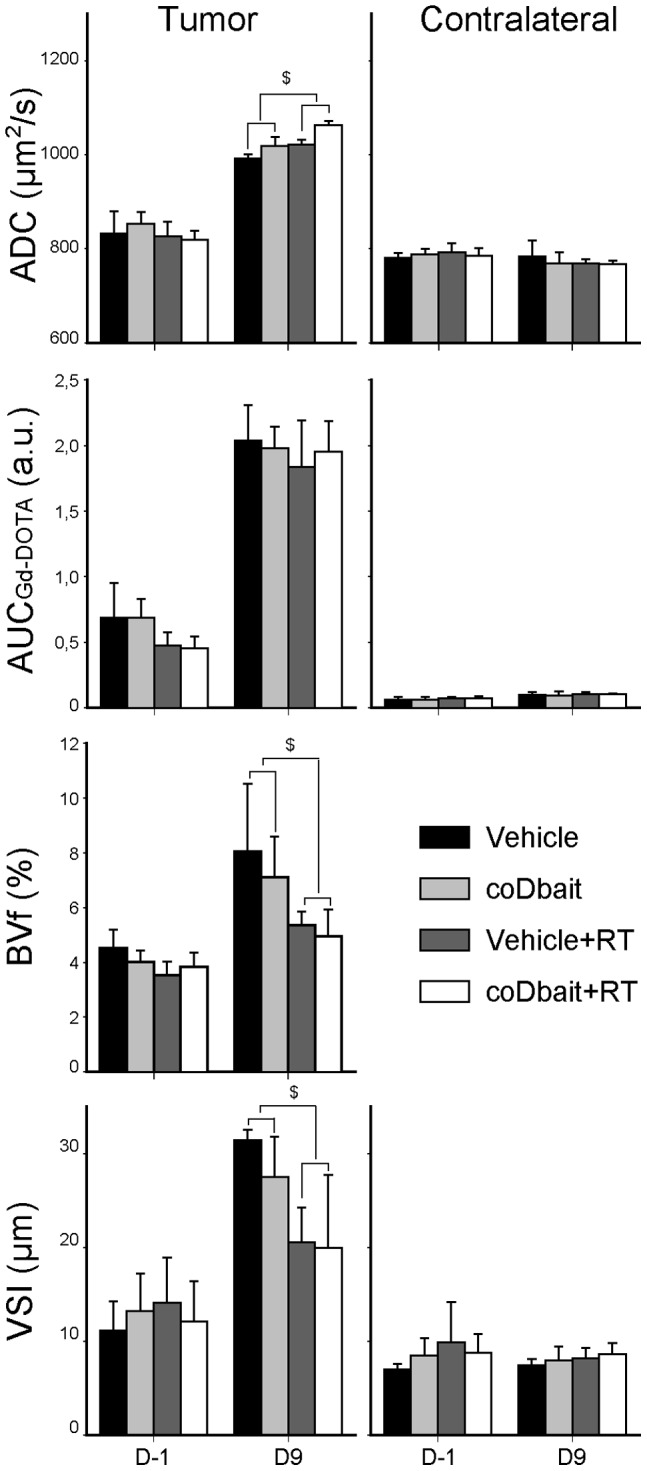
Effect of treatments on tumor properties determined by multiparametric MRI before and after treatment. Multiparametric MRI was performed as given in Fig. 5A. ADC: apparent diffusion coefficient (vehicle n = 6; coDbait n = 6; vehicle+RT n = 9; coDbait+RT n = 10), AUC_Gd-DOTA_: area under curve of Gd-DOTA extravasation (vehicle n = 6; coDbait n = 6; vehicle+RT n = 9; coDbait+RT n = 10). BVf: blood volume fraction. Mean BVf in the contralateral striatum was set at 3% for normalization purposes (vehicle n = 6; coDbait n = 6; vehicle+RT n = 8; coDbait+RT n = 10). VSI: vessel size index (vehicle n = 3; coDbait n = 4; vehicle+RT n = 8; coDbait+RT n = 10). RM-ANOVA, $: effect of irradiation between D-1 and D9, p<0.05. Mean ± SD.

Given that RT is known to promote changes in tumor vasculature, we raised the question of whether coDbait injection associated with RT might additionally modify the tumor microenvironment with an effect on vascularization [30]–[32]. For this purpose, we applied a multiparametric MRI analysis to monitor the vascularization properties of the tumor and the contralateral striatum before and 2 days after the end of treatment (Fig. 5). No differences between treatments were observed in the contralateral striatum for all parameters analyzed. For the vehicle group, all parameters were increased in the tumor between D-1 and D9. RT prevented the augmentation of BVf and VSI found in the vehicle group, whereas it increased the augmentation of ADC in the tumor. The augmentation of AUC_Gd-DOTA_, a measurement that is related to vessel permeability, remained similar for each treatment. Even though a later effect after the end of treatment cannot be excluded, no deleterious or synergic effect of coDbait injection with RT on tumor vasculature was detected 2 days after the end of treatment (D9).

### coDbait with RT Increases ED1-positive Macrophage Infiltration and Destabilizes MBP-Positive Myelin Elements in the Tumor Microenvironment

**Figure 6:**
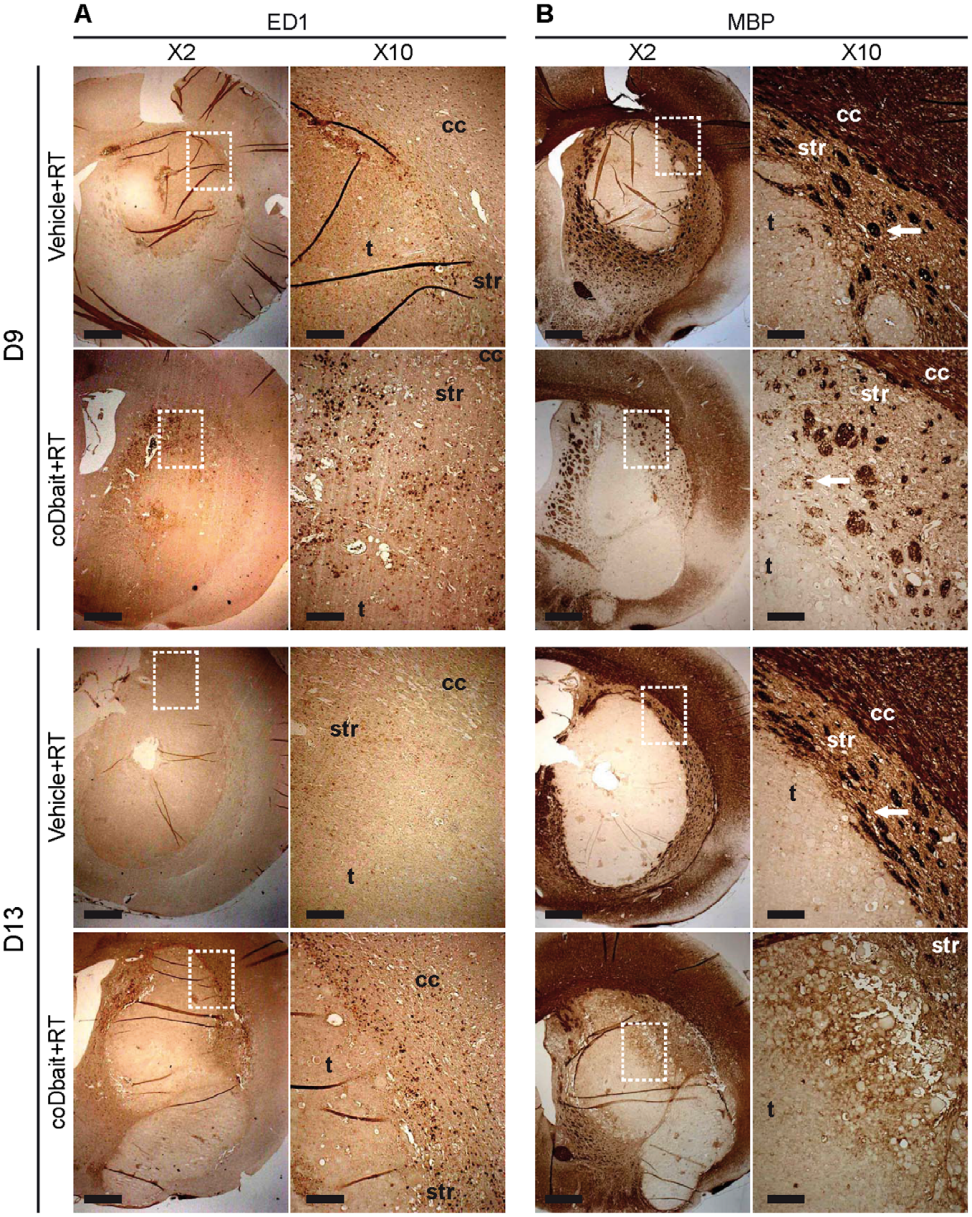
Effect of treatments on myelin sheets and macrophage infiltration before (D-1) and after (D9) treatment. A) Immunodetection of MBP-positive myelin elements. B) Immunodetection of ED1-positive cells. Representative images are presented. D9 (vehicle+RT n = 6; coDbait+RT n = 4) and D13 (vehicle+RT n = 8; coDbait+RT n = 4). Dotted rectangle represents the area of the ×2 magnification; cc = corpus callosum, str = striatum, t = tumor bulk. White arrows in B) show striosomes. ×2 images, scale bar = 1 mm; ×10 images, scale bar = 200 µm.

Two days after treatment completion (D9), immunohistological analysis of ED1-positive macrophages showed a limited infiltration at the tumor border after RT alone (Fig. 6A), whereas many ED1-positive macrophages are detected after coDbait with RT treatment in a wide zone surrounding the tumor. This infiltration decreased on D13. Immunohistological analysis of MBP-positive myelin elements showed that RT alone preserved the structure of striosomes around the tumor (Fig. 6B). At D9, coDbait with RT induced a substantial loss of MBP labeling surrounding the tumor, presumably due to a destabilization of striosomes (Fig. 6B, white arrow). This effect was increased at D13 when striosomes were barely detected in some parts around the tumor.

These observations can be interpreted as a deleterious effect of coDbait with RT on the tumor microenvironment, associated with an increased inflammation accompanied by destabilization of striosomes. This might help reduce the invasion of tumor cells to the surrounding tissues, which is in part assisted by the white matter tract [33].

## Discussion

A therapeutic protocol was designed with two coDbait injections, each followed by a 6-Gy irradiation. CoDbait with RT increased the survival of rats principally by slowing tumor growth, whereas the tumor vasculature remained similar to that of control-irradiated tumors. Here, two cycles of treatment, each consisting of one coDbait injection followed by one irradiation of the head, improved the survival of rats bearing an RG2-glioma. These results are in good agreement with previous studies conducted on human melanoma tumor subcutaneous xenografts [10], [11]. In our study, RT delayed tumor progression at D9, i.e., 2 days after the end of treatment. Interestingly, the effect of coDbait in synergy with RT was observed later, extending tumor growth control to D11 and D13, i.e., 4 and 6 days after the end of treatment. Despite the decrease in cell proliferation at D9 detected with Ki67 labeling, tumor size was similar between both RT groups and the coDbait with RT did not promote changes in the tumor volume *per se.* Such an apparent lack of treatment effect at an early time after treatment has already been well documented clinically by the diagnosis of “pseudo-progression” in patients who respond to treatment [34]–[36].

The multiparametric MRI measurement was used to evaluate whether the synergic effect of coDbait with RT on tumor survival might result from an additional impact on tumor vasculature. BVf, vessel integrity determined with AUC_Gd-DOTA_ and VSI, a value related to vessel diameter, were increased along the natural course of tumor development. At D9, RT reduced BVf and VSI but not AUC_Gd-DOTA_, in agreement with the observed delay in tumor progression. We failed to detect any effects of coDbait or coDbait with RT on tumor vasculature. This is in good agreement with a selective radiosensitzing effect of coDbait strictly on tumor cell proliferation. This result suggests that one additional treatment with antiangiogenic or antivascular drugs that target the tumor vasculature could be used to increase the beneficial effect of coDbait with RT. Such combined therapies are already being investigated in clinical studies [37], [38].

Most previous studies have been conducted on nude mice xenografted with human tumors. Therefore, immune and inflammatory response was not addressed in these studies. Here, for the first time we have described an interesting observation that coDbait with RT increases inflammation and destabilizes the microenvironment. These properties could play an important role in recurrence of glioma.

We show that coDbait amplified the effect of RT by impairing tumor cell proliferation, as seen at D9. The tumor size reduction was no longer detected at D13, i.e., 6 days after the end of treatment. A previous study reported by Quanz et al. [10] showed that upon irradiation, the Dbait molecule exerts a beneficial dose-dependent effect on the life span of mice with a xenografted model of head and neck squamous cell carcinoma. Given the observations of a dose-dependent effect of Dbait molecules, the injection with a larger amount of Dbait molecules would presumably increase the beneficial effect of coDbait with irradiation.

CED induces a pressure-driven flow allowing a homogenous distribution of drugs over large volumes [39]. In our study, coDbait after CED injection does not cover the entire tumor mass. This can be attributed to the high interstitial pressure present in this tumor type, which provokes fluid flow from the tumor to surrounding tissue [40]. The injection volume, i.e., 10 µL, might be insufficient to cover the entire tumor volume. The injected volume might be increased but with the limitations of an extended injection time, limiting its clinical use, where tumors are larger [39].

One alternative to the CED could be the use of an implanted delivery system such as an osmotic pump that can deliver a higher volume of molecules at a constant rate over a long period. This strategy has already shown promising results in the context of glioma for intratumoral delivery of carboplatin [41] and might be suitable in association with a highly fractionated RT that is commonly used in the clinical setting. The local delivery strategy is of great interest because this can be combined in the clinic with resection therapy followed by RT or chemotherapy, thereby causing no further damage from drug delivery [39]. A preliminary study from our group showed that coDbait molecules accumulated in tumor after intravenous injection (data not shown). However, this type of coDbait administration needs further investigation regarding the maximum achievable doses in tumor and systemic toxicity but is highly promising for clinical purposes.

CoDbait molecules have no deleterious effect on healthy brain tissues. The intratumoral injection of coDbait prior to RT improves the survival of rats and reduces tumor volume by amplifying the effect of RT without altering tumor vasculature. CoDbait is therefore a promising molecular therapy to sensitize glioma to RT.
